# Diagnosis and Treatment of Benign Prostatic Hyperplasia in Dogs: New Approaches

**DOI:** 10.1155/vmi/4153172

**Published:** 2025-04-22

**Authors:** Nooshin Derakhshandeh, Asghar Mogheiseh, Saeed Nazifi

**Affiliations:** Department of Clinical Sciences, School of Veterinary Medicine, Shiraz University, Shiraz, Iran

**Keywords:** BPH, diagnosis, dogs, treatment

## Abstract

This review aims to define the best prescription options for the treatment and diagnosis of benign prostatic hyperplasia (BPH) in dogs. BPH has become the most common disease in older dogs. While it rarely poses life-threatening risks, it can significantly impact a patient's quality of life, as changes in prostate size can lead to various clinical signs, including tenesmus, constipation, and hematuria. An accurate initial evaluation and diagnostic method are essential for distinguishing BPH from other conditions and differential diagnoses. Treatment modalities include both medical and surgical options. In recent years, medical therapy for BPH has expanded and improved. The focus of medical treatment is to reduce prostatic volume, alleviate bothersome clinical sign, and prevent the need for surgical intervention, especially for dogs that are still used for breeding. The pharmacological effects, efficacy, and safety of these treatments must be thoroughly analyzed to ensure good drug adherence and persistence.

## 1. Introduction

Benign prostatic hyperplasia (BPH) or PH is a condition commonly observed in intact male dogs and is primarily age-related. This condition is characterized by the proliferation of prostate tissue and an increase in cell volume. The clinical presentation of hyperplasia is dependent on hormonal factors and is marked by an imbalance in the testosterone-dihydrotestosterone (DHT) ratio, which includes a reduction in testosterone concentration alongside an elevation in DHT levels [1]. The metabolites 3*α* and 3β androstenediol, resulting from the degradation of dihydrotestosterone, play a crucial role in the proliferation and hypertrophy of the prostatic glandular epithelium [2]. The concentration of DHT contributes to the enhanced production and expression of prostate growth factors, such as vascular endothelial growth factor (VEGF), which subsequently leads to hyperplasia of the prostatic gland [3]. Furthermore, the hormone prolactin may influence the development of BPH. According to Wolf et al., [4] slight increases in prolactin concentrations within the prostatic secretion were observed as dogs aged and in those diagnosed with BPH. The observed correlations between prolactin levels and testicular steroids suggest a potential role for prolactin in the pathogenesis of canine BPH [4]. Notably, approximately 95% of dogs diagnosed with BPH do not exhibit clinical signs associated with prostatic syndrome [5]. BPH in dogs is characterized by symmetric and asymptomatic hypertrophy, which is typically nonpainful and maintains a normal consistency [3]. Clinical signs may include urethral discharge, hematuria, rectal tenesmus, and constipation. The enlargement of the prostate can lead to edema in the hind limbs, resulting in an unbalanced gait [6]. A study indicated that the clinical manifestations of BPH encompassed straining during urination and defecation (91.2%), urinary incontinence (87.7%), tenesmus (61.4%), hematuria or blood dripping from the penis (45.6%), holding the tail away from the rear (38.5%), weakness (33.3%), weight loss (26.3%), loss of appetite (22.8%), and paralysis of the hind limbs (8.7%) [7].

Another research revealed that the overall occurrence of BPH in dogs stood at 12.8%. The predominant clinical symptoms noted in the affected dogs consisted of passing small, thin, tape-like feces, holding the tail away from the rear, tenesmus, and straining while urinating and defecating. These symptoms were typical of the majority of BPH cases [8].

Additionally, subfertility and hemospermia may manifest even in the absence of generalized clinical signs [6].

A retrospective study involving a cohort of 72,300 male dogs identified 481 cases (0.7%) of prostatic disorders through clinical symptomatology and ultrasonographic assessment. The most commonly diagnosed conditions included BPH (45.9%) and prostatitis (38.5%), followed by prostatic abscesses (7.7%), cysts (5.0%), neoplasia (2.6%), and squamous metaplasia (0.2%). The study further elucidated that distinct prostatic conditions were associated with specific clinical presentations: Urinary symptoms (e.g., dysuria, hematuria) were observed in more than 50% of cases diagnosed with prostatitis, prostatic abscesses, and cysts. Systemic symptoms (e.g., lethargy, fever, weight loss) were noted in 28% of dogs with prostatic abscesses and in 46% of those with prostatic tumors. Genital symptoms (e.g., abnormal discharge, testicular abnormalities) were prevalent in cases of prostatitis (45%), BPH (53%), and prostatic tumors (82%). Gastrointestinal symptoms (e.g., constipation, tenesmus) were documented in 75% of cases involving prostatic abscesses and in 82% of dogs with tumors, indicating a potential mechanical impact of prostatic enlargement on rectal functionality [9].

A 9-year retrospective investigation has revealed that clinical signs demonstrate varying degrees of association with specific prostatic disorders in dogs. The results emphasize critical predictive markers that may facilitate the differentiation between benign and pathological conditions, thereby improving diagnostic precision in veterinary practice. Cazzuli et al. [10] identified BPH as the most commonly diagnosed prostatic disorder (71.6%), followed by prostatitis (23.5%), with prostatic neoplasia being relatively rare (3.1%). The mean age of affected dogs was 10 years, with cases occurring in individuals aged 3–15 years. Large-sized dogs constituted the majority of cases (62%), followed by medium-sized (27%) and small-sized dogs (11%). Furthermore, 99% of affected dogs were noncastrated, underscoring the role of testosterone in the pathogenesis of prostatic disorders. To systematically evaluate clinical manifestations, the study classified symptoms into three principal categories: Digestive signs: Tenesmus, anorexia, weight loss, constipation, diarrhea, hematochezia. Urinary signs: Hematuria, dysuria, urinary incontinence, polyuria/polydipsia. Other signs: Lethargy, prostatomegaly, perineal hernia, abdominal pain. Among these, tenesmus (34%) was the most frequently observed symptom, though it was considered nonspecific. Other prevalent signs included anorexia (32%), lethargy (27%), prostatomegaly or pain upon rectal examination (25%), and abdominal pain (22%). Notably, weight loss (19%) was more frequently associated with neoplastic conditions, highlighting its potential as a strong predictive indicator of malignancy. Moreover, the findings suggested that hematochezia was more commonly linked to BPH than prostatitis, possibly due to rectal compression caused by prostatic enlargement. Urinary signs were less frequently observed in BPH but were more prominently associated with prostatitis, particularly hematuria and urinary incontinence [10].

An important area of recent interest in veterinary medicine is the diagnosis of prostatic disorders, which is driven by the increasing attention that pet owners are giving to their animals' health, along with the enhanced availability of diagnostic tools and therapies [11]. The diagnosis of BPH in dogs is typically presumptive, relying on physical examination, patient history, ultrasonographic findings, clinical signs, fine needle aspirate, prostatic wash, or monitored response to therapy [2]. However, the vast majority of dogs affected by BPH does not exhibit and will not exhibit clinical signs. Furthermore, similar to humans, there is no direct correlation between prostate size and the severity of BPH with clinical signs [12]. In recent years, extensive research has been undertaken to identify prostatic disorders as early as possible. Techniques such as measuring serum concentrations of canine prostate-specific esterase (CPSE), cytological evaluation of prostatic fluid, and, in some cases, advanced imaging methods like computed tomography (CT) may prove beneficial [13]. Accurate diagnosis is a critical factor in making appropriate treatment decisions. Although clinical signs and examination findings may be similar across different conditions, the treatment strategies can vary significantly [14]. Treatment for BPH primarily focuses on reducing the size of the prostate gland. Presently, castration is considered the most effective approach for decreasing prostate size in dogs [15]. For those not intended for breeding, surgical castration is the preferred treatment method. Complete involution of the gland generally occurs within 6–12 weeks after gonadectomy, although clinical signs may diminish earlier [16]. In breeding dogs, pharmacological treatments that inhibit the production or action of androgens may be preferred to maintain fertility. The treatment strategy involves the suppression or prevention of androgen synthesis or action [3]. However, the pharmacological treatment for BPH should be avoided in cases where testicular tumors are also present. It is crucial to exclude the possibility of testicular tumors before initiating antiandrogen therapy, as these tumors may produce estrogens. In conditions dependent on estrogens, such as prostatic metaplasia, antiandrogen therapy would be both ineffective and inappropriate [17]. This review article aims to explore innovative diagnostic methods and effective treatments for the most common prostatic disease in dogs, BPH.

## 2. Diagnosis and Diagnostic Evaluation

### 2.1. Clinical Signs and Digital Rectal Palpation

The diagnosis of symptomatic BPH generally includes a physical examination via clinical sign and palpation, which shows that the prostate is symmetrically enlarged and nonpainful. During a digital rectal examination, the prostate may appear enlarged, symmetrical, mobile, of usual consistency, and not tender [8].

The assessment of prostatic disorders involves a thorough medical history; a digital rectal examination to determine the size, shape, and consistency of the prostate, as well as to check for any discomfort, keeping in mind that a significantly enlarged prostate might be situated in the abdomen, making it challenging or even impossible to evaluate through rectal examination [7].

Digital palpation is a simple and cost-effective procedure that can be conducted during a standard general examination [12].

The investigation of one study was focused on the histological occurrence of subclinical prostatic diseases in mixed-breed male dogs and assessed the effectiveness of digital rectal examination as a screening tool. Rectal examination was conducted on 500 dogs. The total occurrence of subclinical prostatic disease was found to be 75.6%. The most frequently observed conditions included BPH (44.8%), prostatitis (23.6%), and prostatic neoplasia (3.6%). DRE demonstrated a high specificity (75%), and positive predictive value (87%) [18].

Another research that was concentrated on subclinical BPH in male dogs. It included a sample of 65 male dogs, with 35 identified as having subclinical BPH and 30 serving as a healthy control group. The findings indicated that early identification of subclinical BPH is achievable through a combination of assessments, including alterations in ultrasound characteristics, CPSE levels, and rectal examinations [12].

Rectal examination allows practitioners to evaluate the position and symmetry of the prostate gland. However, it is not considered a highly accurate examination method. Additionally, a rectal examination may not detect mild prostatomegaly if the prostate remains within the pelvic canal [19]. This method also can create difficulties in small dogs due to their size or in giant breeds where the prostate may be located too far cranially [15].

Despite these constraints, it is advisable to incorporate this examination into standard protocols for dogs whenever feasible, as it can provide essential direction for additional inquiries if needed [15].

Intermittent bloody discharge from the prepuce without urination is one of the most frequently clinical signs. Hematuria often occurs mainly at the end of urination [6]. The enlarged prostate may put pressure on the colon, resulting in constipation, tenesmus, or intermittent diarrhea. Severe constipation can also lead to the formation of a perineal hernia [20]. Hematospermia, occurring without changes in semen quality, is the most common symptom during the early stages of BPH. In the later stages, there is a noticeable decline in motility and morphology, along with an increase in tail abnormalities [21].

### 2.2. Imaging Techniques

Ultrasound is a widely utilized diagnostic imaging technique that can identify changes in echogenicity, structure, size, and shape of the prostate, as well as focal lesions within its parenchyma [2]. The prostate glands in dogs with BPH mainly displayed symmetrical shapes and heterogeneous tissue structures, characterized by a diffuse cystic pattern. In comparison, healthy dogs exhibited different results, primarily demonstrating homogeneous tissue patterns and an absence of intraprostatic cysts [22].

A study by Nizanski et al. [23] involving 10 dogs diagnosed with BPH, with a mean age of 9.5 years and an average weight of 14.12 kg, reported similar ultrasonographic findings. In this group, the prostate gland was found to be heterogeneous, featuring focal lesions measuring less than 1 cm in diameter, identified as intraprostatic cysts [23]. Likewise, Russo et al. [24] observed comparable results in a study of eight dogs (mean age 5.9 ± 3.2 years and weight ranging from 19 to 37 kg) diagnosed with BPH, which displayed increased tissue echogenicity and the presence of intraprostatic cysts in all cases [24]. Additionally, a study by Laurusevičius et al. [12] involving 35 dogs with subclinical BPH, with a mean age of 6.9 years and an average weight of 40.1 kg, revealed that the prostate exhibited symmetrical shapes and heterogeneous tissue structures, characterized by a diffuse cystic pattern in dogs with subclinical BPH [12]. Several studies have suggested formulas to assess the size of the prostate gland in both normal and BPH-affected dogs, considering factors like age and weight, to establish the optimal age for performing preventive ultrasonographic examinations of the prostate. Prostatic dimensions are measured as the formula proposed by Ruel et al. [25] and Francey [26]: Volume (cm^3^) = (L (prostatic length) × W (prostatic width) × H (prostatic height) × 0.523 and Volume [cm^3^] = (0.867 × BW [kg]) + (1.885 × age [year]) + 15.88, respectively [25, 26]. In a study conducted by Pasikowska et al. [27]; CT served as an effective instrument for identifying prostate conditions, such as BPH, by analyzing heterogeneity, density, and prostatic metrics. The results revealed the prostatic measurements of BPH revealed a length of 43.87 mm ± 11, a width of 48.95 mm ± 8.76, and a height of 44.9 mm ± 9.48 in the dogs that were affected [27]. In the study conducted by Hosseinpour et al. [28]; 24 intact male dogs with an average age of 7.6 years and an average weight of 13.6 kg, all showing clinical signs related to BPH, were examined using ultrasound to calculate their prostatic volume. The mean prostatic volume was determined to be 14.32 ± 12.62 cm^3^ [28]. In another study conducted by Khaki et al. [29]; the dimensions of the prostate gland in 10 intact dogs diagnosed with BPH were evaluated using sonography. The dogs had an average weight of 8.91 ± 2.5 kg and were over 5 years old. The findings indicated an average prostatic length of 45.78 ± 4.78 mm, a width of 45.78 ± 4.78 mm, and a volume of 41.5 ± 3.05 cm^3^ [29]. Different findings also support the notion that prostate gland volume is greater in dogs affected by BPH compared to healthy dogs, as indicated in recent studies. In the study conducted by Derakhshandeh et al. [30]; 25 intact male mixed-breed dogs induced with BPH, aged between 1 and 3 years and weighing between 15 and 20 kg, were assessed using ultrasonography. The results showed a significant increase in prostate volume in the induction group, rising from 9.66 ± 4.81 cm^3^ on day 0–20.59 ± 6.83 cm^3^ by day 63 [30]. Laurusevičius et al. [12] identified differences in prostatic dimensions between dogs with subclinical BPH and those in the healthy group. The measurements for the subclinical BPH group included a prostatic length of 5.34 ± 1.29 cm, a width of 5.05 ± 1.17 cm, and a height of 4.12 ± 0.94 cm. In contrast, the healthy group had measurements of 3.57 ± 0.77 cm for length, 3.70 ± 0.94 cm for width, and 3.45 ± 0.88 cm for height. Additionally, the data revealed that the subclinical BPH group had a larger prostatic volume, measuring 64.51 ± 43.62 cm^3^, compared to the healthy group, which had a prostatic volume of 26.93 ± 17.93 cm^3^ [12]. In one study, ultrasonographic images of the canine prostate were examined during the induction of BPH in five dogs over 63 days using image analysis software (Image J software, Texture Analysis plugin). The primary focus of the analysis was the echotexture of the prostate parenchyma. However, there were no significant changes observed in the echotexture of the prostate parenchyma throughout the 63-day BPH induction period. The findings suggested that no alterations occurred in the echotexture during the early stages of BPH, although some changes in prostate size were noted. The authors concluded that the dogs did not exhibit any clinical signs of BPH during or at the end of the induction period and that the analysis of prostatic parenchymal echotexture using the software cannot be considered a reliable diagnostic method for BPH in dogs during its early stages [31]. However, ultrasound findings are often not specific to any particular disease and may occasionally overlap with symptoms of other conditions, squamous metaplasia, prostatitis, prostatic cysts and abscesses, and prostatic neoplasia, due to similarities in their etiology and pathology [12, 32]. Doppler evaluation is a noninvasive diagnostic tool that can be readily implemented in clinical practice. In dogs diagnosed with BPH, it indicates a significant increase in blood flow velocity within the prostatic artery [33].

This technique provides comprehensive information regarding the vascularization of the prostate gland, making it valuable for the early detection of prostate diseases, differentiating between malignant and benign lesions, and staging conditions that have already been diagnosed [12, 17, 34]. Elastography evaluates the presence of deformities and assesses tissue stiffness [35, 36].

One research study assessed the application of elastography in identifying prostatic changes in dogs. The study included 22 healthy canines and 45 with diagnosed prostatic conditions, identified through elastographic techniques. All healthy prostates exhibited a uniform hard parenchyma. In contrast, only 35.5% of affected prostates displayed a uniform hard parenchyma, while 62.2% presented a heterogeneous hard parenchyma, and one case showed a soft homogeneous parenchyma. The researchers concluded a significant correlation between elastographic heterogeneity and the presence of disease, with a sensitivity of 64.44% and a specificity of 100% [37].

A research investigation aimed to examine the correlation between elastography and canine prostatic serum esterase in the diagnosis of prostate disorders in canines. The prostate was found to exhibit intermediate stiffness in 84.7% of the left lobes and 79.27% of the right lobes, which was softer compared to the abdominal wall. They posited that, although no linear correlation was established between CPSE and elastography values, the analogous elastographic patterns observed in healthy prostates indicate that elastography may prove beneficial for noninvasive diagnosis of prostatic disorders, thereby enhancing diagnostic precision [38].

Although there are a limited number of experimental studies on this technique, it seems to serve as an effective diagnostic tool for distinguishing between a normal prostate and one that is pathological.

Recently, CT has become an important diagnostic imaging technique in veterinary medicine, helping to minimize structural overlap during examinations of the reproductive system and the caudal abdomen. CT allows for a thorough evaluation of the prostate and adjacent pelvic structures, as well as the assessment of parenchymal and perfusion parameters [39]. Several studies have emphasized the essential role of CT in diagnosing canine BPH. The prostate gland in BPH-affected and healthy intact male dogs was assessed using ultrasonography and various CT techniques. In this study, 52 intact male dogs of different breeds, ages, and weights were categorized into two groups: a healthy group (*n* = 24) and a subclinical BPH group (*n* = 28).

The findings revealed that the length, width, height, and volume of the prostate gland were significantly larger in the BPH-affected group compared to the healthy group. Additionally, dogs with BPH exhibited lower contrast attenuation values and higher ratios of prostate gland dimensions to the sixth lumbar vertebra in comparison to healthy dogs. The ratios of prostate gland width and height to pelvic inlet dimensions were also increased in the BPH group. Ultimately, the authors concluded that utilizing multiple CT imaging methods offers reliable advanced imaging of the prostate gland and serves as an effective diagnostic tool for BPH in dogs [40]. In one study, prostatic perfusion was evaluated using contrast-enhanced ultrasonography (CEUS) and CT perfusion (CTP), along with assessing the CT volumes of the prostate in normal dogs and those with mild and severe BPH before and after castration. The results from both CEUS and CTP revealed that prostates affected by BPH exhibited significantly slower arterial inflow and relatively faster venous outflow compared to normal prostates. Furthermore, blood volume and all perfusion parameters within the prostate showed a significant decrease by day 60 after castration [41]. Genov and Ivanova [14] asserted that the application of imaging techniques allows for accurate differentiation of BPH from other conditions, enabling timely preventative measures and treatments. In their study, two hundred fifty patients of various breeds were evaluated, with 80 having a prior clinical history and complaints related to BPH, and ages ranging from 3 to 7 years. The results from echographic studies showed that 72% of the patients displayed signs of hypo to hyper-heterogeneous parenchyma with moderate heterogeneity, which are characteristics commonly associated with BPH. Additionally, the findings indicated that CT revealed structural changes and provided precise measurements of prostate size, demonstrating that CT is more accurate than sonography. Moreover, an urgent diagnosis is often required in these cases [14]. Vali et al. [39] explored the relationship between CT findings and cytological results in the assessment of the canine prostate. In their study, 30 mature male dogs underwent both cytological examination and CT imaging. The findings revealed that 11 out of the 30 dogs (36.66%) were diagnosed with BPH. They concluded that there is a moderate level of agreement between the cytological results and the final diagnosis based on pre-contrast and post-contrast CT images. Furthermore, no correlation was found between parenchymal heterogeneity and the final fine-needle aspiration (FNA) outcomes. This heterogeneity may result from aging and the formation of fibrous tissue, which can affect tissue structure and blood supply [39]. According to Rodak et al. [42]; the signs of prostatic diseases in dogs are often nonspecific. Effective treatment should rely on a thorough investigation using dependable diagnostic tools. In their study, they assessed the diagnostic value of ultrasonography and FNA cytology in canine prostatic diseases. The mean accuracy of FNA cytology was found to be 0.72, while ultrasonography demonstrated a higher accuracy of 0.88. They concluded that while the diagnosis based on ultrasonography is highly reliable, it should be complemented with clinical signs [42].

Pasikowska et al. [27] noted that CT scanning has specific limitations. Differentiating between the prostate capsule and parenchyma is not feasible in CT images. In contrast to an ultrasound examination, CT scans are less accessible, more costly, time-consuming, and often necessitate general anaesthesia. While ultrasonographic measurements of the prostate may not be as accurate as those from CT scans, they are quick and noninvasive. It is improbable that CT will supplant ultrasound examinations for the prostate, particularly in diagnosing prostate hyperplasia. Nevertheless, this imaging technique should be considered in instances where there is uncertainty regarding the interpretation of ultrasound findings [27].

### 2.3. Cytological and Pathological Analysis

Cytological and histopathological evaluation of prostate gland material can be conducted to differentiate or confirm a presumptive diagnosis [42]. Three techniques for collecting prostatic cytological material have been described: ejaculation by manual stimulation, prostatic wash, and FNA. Ultrasonography-guided FNA is a quick and simple method [43]. In a specific investigation, the results of prostatic material cytology were compared with histopathological findings in canines diagnosed with clinically evident BPH, demonstrating an 80% concordance in diagnostic outcomes regardless of the method employed for specimen collection. Specifically, for the ultrasonography-guided FNA technique, the alignment rate was 75% [44]. The results from Rodak et al. [42] indicated that the mean accuracy, sensitivity, specificity, and precision for FNA cytology compared to histopathological findings were 0.72, 0.67, 1.00, and 0.83, respectively. In contrast, when comparing ultrasonography results with histopathological findings, these values were 0.88 for accuracy, and 0.78 for sensitivity, and both specificity and precision were 1.00. They recommended that ultrasonography findings, when combined with clinical signs, should be regarded as reliable for a definitive diagnosis, except in cases where neoplasia is suspected. They also affirmed the high diagnostic value of FNA cytology in diagnosing prostatic diseases. FNA cytology was considered a highly accurate method for diagnosing BPH and prostatitis, particularly in situations where clinical signs and ultrasound findings did not provide a conclusive diagnosis [42].

### 2.4. CPSE as a Specific Serum Biomarker

Serum CPSE has been recognized as a valid and specific biomarker for canine prostatic disorders, as its levels are elevated in the serum of dogs suffering from various prostatic conditions, including BPH, bacterial prostatitis, and prostate cancer [6, 11, 45]. Indeed, the assessment of CPSE serves three distinct purposes in the and rological clinical examination: first, it acts as a diagnostic tool for identifying prostatic disorders [11, 45]; second, it functions as a biomarker for the early screening of prostate health in dogs [46]; and third, it aids in the follow-up of patients suffering from prostatic diseases who are undergoing medical treatments [47]. The determination of CPSE levels does not eliminate the need for additional complementary tests to rule out the presence of other prostatic diseases, such as prostatitis, squamous metaplasia, or neoplasia. These additional tests are particularly recommended when clinical examination and/or ultrasonography suggest disorders other than BPH [45]. In one study involving 79 dogs that were monitored after examination, the development of clinical signs was significantly more prevalent in dogs with a relative prostate size of ≥ 2.5 at baseline (*n* = 20) compared to those with smaller prostates (*n* = 51). The results demonstrated the utility of CPSE for estimating both the current and future prostatic size in dogs aged 4 years and older. However, the concentrations of CPSE did not differ between dogs that had developed clinical signs and those that had not done so. Therefore, the findings highlighted the clinical relevance of prostatic size in predicting the emergence of clinical signs associated with prostatic disease in dogs [45]. In one study involving 79 dogs that were monitored after examination, the development of clinical signs was significantly more prevalent in dogs with a relative prostate size of ≥ 2.5 at baseline (*n* = 20) compared to those with smaller prostates (*n* = 51). The results demonstrated the utility of CPSE for estimating both the current and future prostatic size in dogs aged 4 years and older. However, the concentrations of CPSE did not differ between dogs that had developed clinical signs and those that had not done so. Therefore, the findings highlighted the clinical relevance of prostatic size in predicting the emergence of clinical signs associated with prostatic disease in dogs the result of this study demonstrated that combining CPSE analysis with ultrasonography enhances early diagnostic accuracy while minimizing reliance on expensive equipment [48].

In contrast, Golchin-Rad et al. [49] argued that there is a positive correlation between prostate volume and CPSE concentrations during the induction of prostatic hyperplasia in dogs [49]. Moreover, another study [46] found that healthy dogs had average CPSE values of 38.9 ng/mL, while dogs with BPH had values averaging 184.9 ng/mL. In this study, an ultrasonographic examination of the prostate was performed on 19 dogs (weighing between 6 and 40 kg; aged 1–5 years) that showed no symptoms of prostatic diseases. The dogs were divided into two groups: Group A, which included 11 dogs without prostatic disorders, and Group B, consisting of 8 dogs that exhibited prostatic disorders on ultrasound, characterized by altered appearance, the presence of cysts, or irregular borders. The study assessed the concentration of CPSE and the ratio between the calculated and expected normal prostatic volume for each dog. It was found that the estimated volume ratio was greater than 1.5 in dogs from Group A. These findings suggest that a CPSE serum concentration exceeding 50 ng/mL in asymptomatic dogs is linked to ultrasonographic changes and increased prostatic size (with a volume 1.5 times greater than normal). Given that the onset of prostatic disorders often remains asymptomatic, the rapid assessment of CPSE could be advantageous for identifying animals that may need further evaluation [46]. Pinheiro et al. [45] suggested that, due to the nonspecific nature of clinical and ultrasonographic findings in cases of BPH, measuring CPSE in hyperplastic dogs could be advantageous. They assessed CPSE levels in both negative controls and hyperplastic dogs, utilizing cytological findings as the reference method. Notably, the control group comprised middle-aged intact dogs (with a median age of 5.0 years), which differs from previous studies that employed very young control dogs. The data revealed significant differences in median CPSE levels when comparing controls to hyperplastic dogs (29.1 ng/mL versus 160.7 ng/mL, respectively). Additionally, significant positive correlations were found between median CPSE levels and both age and prostatic volume. The study indicated agreement between CPSE levels and cytological results. They concluded that measuring CPSE is a useful and accurate method that should be considered as an alternative or complementary tool to conventional methods for diagnosing BPH in middle-aged dogs [45]. One study aligns with prior research [45], researchers examined significant differences in serum CPSE levels between healthy dogs and those affected by subclinical BPH. The healthy group showed a mean serum CPSE level of 38.85 ± 14.55 ng/mL, with values ranging from 17.53 to 67.8 ng/mL. In contrast, dogs diagnosed with BPH had a markedly higher mean CPSE level of 203.3 ± 90.39 ng/mL, with levels ranging from 97.31 to 487.54 ng/mL [12].

### 2.5. New Diagnostic and Prognostic Markers

Numerous specific biomarkers with prognostic and diagnostic values that are comparable to histopathological findings are currently utilized for the noninvasive diagnosis of prostate diseases in humans. MicroRNA is a single-stranded, noncoding RNA molecule that ranges from 21 to 25 nucleotides in length and plays a role in regulating various gene expressions. Increased levels of miRNA can be found in systemic circulation following tissue injury, indicating that miRNA is released as a result of cell damage. Because of their remarkable stability, ease of detection in physiological fluids, and specific expression patterns in tissues, miRNAs have emerged as specific biomarkers for organ damage and cancer development [50]. VEGF is a protein recognized as one of the key mechanisms involved in angiogenesis and the increase in blood vessel permeability. Numerous studies have demonstrated that excessive activation of VEGF can result in hypertrophic processes or carcinogenesis of the prostate gland [51, 52]. Wieszczeczyński et al. [53] assessed the effectiveness of two biomarkers, miRNA-129 and VEGF, in diagnosing BPH in dogs. In their study, 40 dogs were categorized into three groups: Group I included healthy dogs up to 5 years old, Group II consisted of dogs aged 5–10 years with BPH, and Group III comprised dogs over 10 years of age with confirmed BPH. The results showed that dogs in Groups II and III exhibited a significant decrease in miRNA expression and a notable increase in serum VEGF levels compared to dogs in Group I. Furthermore; a positive correlation was identified between prostate size and VEGF levels. These findings suggest that miRNA-129 and VEGF could play a significant role in diagnosing prostatic disorders in dogs [53]. However, the study conducted by Kobayashi et al. [54] found that analyzing miRNA expression in both tissues and body fluids of healthy dogs and those with BPH did not show significant changes in miRNA levels. In contrast, notable alterations in expression were identified in cases of prostate cancer. The authors suggested that the differences in expression levels are mainly due to the varying types of miRNA molecules used in diagnostic testing [54]. Further research into the roles of miR-129 and VEGF should be conducted, as the resulting findings could enhance our understanding of noninvasive diagnosis for prostate diseases in dogs. This exploration may lead to improved diagnostic methods and better management of prostatic disorders in veterinary medicine.

## 3. Management of BPH Patients

### 3.1. Castration

Treatment for BPH focuses on suppressing or preventing the synthesis or action of androgens [17]. Castration is the most effective way to reduce prostate size in canines. Castration can decrease prostate size by as much as 70%, but it may take up to 4 months for the full effects to be realized. The drawbacks of castration include the potential for surgical and anesthetic complications, as well as the resulting sterility in breeding dogs. For breeding animals or dogs with preexisting prostatic bacterial infections, alternative approaches, such as medical management, may be taken into account [16]. The study evaluated the effects of orchiectomy on hormonal profiles, prostate apoptosis, blood flow, and biometry in dogs suffering from BPH. A 67.4% percentage reduction in prostatic volume was noted in dogs that underwent orchiectomy 60 days after the procedure. On day 60, the orchiectomized groups demonstrated increased prostatic blood flow compared to the untreated groups. Moreover, these groups exhibited a rise in prostate artery resistance. Orchiectomy also led to a significant decrease in androgen concentrations 30 days post-castration. They assumed that gonadectomy promoted marked prostate atrophy, causing extensive tissue regression, and ultimately decreasing overall prostatic gene expression. Gonadectomy resulted in prostate atrophy and significant endocrine alterations in dogs with BPH [55]. Another study believed that orchiectomies dogs experienced a significant reduction in prostate volume, primarily due to a notable decrease in testosterone and dihydrotestosterone concentrations shortly after the procedure. Surgical intervention can lead to a 50% reduction in prostatic volume within 3 weeks post-orchiectomy, ultimately resulting in prostate atrophy [56]. Castration serves as a swift, efficient, and enduring solution for dogs suffering from BPH, leading to a quick reduction in blood testosterone levels and gland size, all while leaving serum oestradiol levels unaffected. One research study investigated the impact of castration on the dimensions of the prostate as well as the alterations in serum levels of testosterone and oestradiol in both healthy dogs and those suffering from BPH on days 0, 7, 14, 28, 60, and 90 following castration. The findings indicated that castration had no significant effect on serum estradiol levels. Conversely, castration resulted in a reduction of serum testosterone (with values falling below 20 ng/dL in the first week), leading to a gradual decrease in prostate size in relation to body weight [57]. A review article focused on the diagnosis and management of BPH revealed that the most effective treatment for canine BPH is castration, which can lead to a 70% reduction in prostate size within 7–14 days following the procedure [58].

### 3.2. Androgen Receptor Antagonists

Androgen receptor blockers like flutamide, and osaterone acetate, progestins such as megestrol acetate (MGA), medroxyprogesterone acetate (MPA), chlormadinone acetate (CMA), and delmadinone acetate (DMA) and gonadotropin-releasing hormone (GnRH) agonists like deslorelin acetate, buserelin, nafarelin, leuprolide, deslorelin and goserelin are treatment options available for managing conditions related to androgen activity in dogs. These medications can regulate hormonal levels and mitigate the effects of androgens on the prostate. Oral administration of MGA at a dosage of 0.5 mg/kg/day for 2 months has been shown to reduce prostatic size within 1-2 months after initiating treatment [59].

MPA, administered at 3-4 mg/kg body weight via subcutaneous injection every 5 months, leads to a decrease in testosterone levels starting in the fifth week of treatment, resulting in prostate shrinkage within 4–6 weeks in 53% of cases and resolution of clinical signs in 84% of BPH cases [60, 61].

A 5-month oral treatment with CMA at a dosage of 0.3 mg/kg body weight effectively reduced prostatic size without affecting testicular or pituitary function [59].

Osaterone acetate is an effective and potent competitive inhibitor of testosterone receptors. It hinders the uptake of dihydrotestosterone in the prostate gland and inhibits the activity of the enzyme 5α-reductase (5α-R). Additionally, osaterone acetate reduces the levels of dihydrotestosterone and androgen nuclear receptors in the prostate. It has a regressing effect on the prostate gland that is five times stronger than that of CMA. However, a slight decrease in serum testosterone levels has been observed in animals treated with osaterone acetate indicating that a minor antigonadotropic effect cannot be entirely ruled out [62]. Murakoshi et al. [63] noted a temporary decrease in testosterone levels during treatment with osaterone acetate [63]. Similarly, Tsutsui et al. [64] also reported comparable findings concerning the reduction in testosterone levels during osaterone acetate treatment [64].

A randomized clinical trial was conducted to compare the efficacy and therapeutic profiles of osaterone acetate and deslorelin acetate in male dogs exhibiting clinical signs of BPH. The dogs were treated with either osaterone acetate or deslorelin acetate. The results indicated that both osaterone acetate and deslorelin acetate were effective in treating BPH, achieving clinical remission in all treated dogs. However, complete alleviation of BPH symptoms occurred more rapidly with osaterone acetate, as 80% of dogs showed improvement by day 7, compared to 40% of dogs treated with deslorelin acetate, which experienced relief within the first 21 days. Clinical signs of BPH recurred in dogs treated with osaterone acetate starting from week 24, while no relapses were observed in dogs treated with deslorelin acetate by the end of the 36-week observation period. In conclusion, both osaterone acetate and deslorelin acetate were found to be effective and safe treatment options for managing BPH-related symptoms in dogs [23]. Albouy et al. [65] observed that most of the dogs experienced complete remission of clinical signs throughout the 6-month trial period, although 16% of the dogs exhibited clinical relapse and an increase in prostate volume during follow-up visits. Nonetheless, it is important to highlight that both osaterone acetate and deslorelin acetate were effective in reducing clinical signs. Dogs treated with osaterone acetate showed a quicker response (by week +2) without experiencing a flare-up effect, whereas dogs treated with deslorelin acetate demonstrated improvement later (between week +3 and week +8) [65]. In adult dogs treated with an implant containing 0.5–1.0 mg deslorelin per kg of body weight, serum testosterone concentrations showed a significant decrease. The decline in blood testosterone levels is also associated with a measurable reduction in the size of the prostate gland [66]. In a study involving seven dogs, the administration of implants containing 6.6 mg of buserelin resulted in a reduction of testosterone and estradiol concentrations to baseline levels within 15 days. Additionally, both testicular and prostatic sizes were reversibly diminished [62, 67]. Other GnRH agonists, such as implants containing azagly-nafarelin, have been shown to reduce prostatic size and testicular size by 52% and 54% after 8 weeks of treatment, respectively [68]. Additionally, BPH quickly resolved following the administration of the implant. Nizański et al. [23] proposed that using a 4.7 mg deslorelin implant in conjunction with osaterone acetate at a dosage of 0.25–0.5 mg/kg is highly effective, resulting in a significant and sustained decrease in prostate gland volume for at least 5 months [23]. D'Francisco et al. [34] investigated the effects of a single dose of the third-generation GnRH antagonist, acyline, on canine BPH. In this study, seven mixed-breed dogs diagnosed with BPH received acyline at a dosage of 330 mg/kg via subcutaneous injection on day 0. The dogs were then evaluated through ultrasound on days 15, 30, and 60 following treatment. By day 30, prostatic volume had decreased by an average of 38.44%, and the mean echogenicity and heterogeneity of the prostatic parenchyma improved in all post-treatment evaluations. Additionally, pretreatment intraprostatic cysts were found to have disappeared at the peak treatment effect. The resistance index of the prostatic arteries showed an increase on day 30, differing from values recorded on day 7 and day 60. The study concluded that a single administration of a third-generation GnRH antagonist safely reduced prostatic volume, parenchyma, and blood flow abnormalities associated with canine BPH over 30 days. Monthly administrations of this treatment could provide a rapid, effective, and safe therapeutic option for managing BPH [34]. GnRH antagonists have not been widely introduced into veterinary practice mainly due to the high costs of the drug and the necessity of relatively frequent administration [17].

### 3.3. 5α-R Inhibitor

5α-R inhibitors are essential in preventing the conversion of testosterone to dihydrotestosterone. By blocking this enzymatic process, these inhibitors contribute to a reduction in the size of the prostate gland. 5α-R inhibitors can be divided into two categories based on their mechanisms of action: competitive inhibitors, such as finasteride, and noncompetitive inhibitors, like epristeride [69]. Competitive 5α-R inhibitors were found to significantly decrease dihydrotestosterone levels in peripheral blood which subsequently led to an increase in testosterone levels in both the bloodstream and the prostate gland [70]. One study examined the effects of BPH and finasteride therapy on the hemodynamic and vascular characteristics of the canine prostate. Dogs undergoing BPH-finasteride treatment were assessed using ultrasonography of the prostatic artery at monthly intervals (on days 0, 30, and 60) to measure prostate volume, expected prostate volume, prostate vascularization score, and prostatic artery blood flow parameters. The results indicated a decrease in the prostate vascularization score from day 0 to day 60 in the treated dogs. Additionally, day 60 found no significant difference between the actual prostate volume and the expected prostate volume. The researchers concluded that finasteride treatment effectively reduces prostate volume, local vascularization, and blood flow, making it a valuable option for managing BPH [71]. Lima et al. [55] investigated the effects of different doses of finasteride and orchiectomy on hormonal profiles, prostate apoptosis, blood flow, and biometry in dogs with BPH. The dogs were divided into several groups: untreated, those receiving 0.1 mg, 0.2 mg, or 0.5 mg/kg/day of finasteride, along with a group that underwent orchiectomy. The results showed that by day 60, the percentage reduction in prostatic volume was more pronounced in the orchiectomized dogs compared to those treated with finasteride, with finasteride also proving more effective than the untreated group. On day 60, the groups receiving 0.2 and 0.5 mg/kg/day of finasteride, as well as the orchiectomy group, demonstrated higher prostatic blood flow than the group receiving 0.1 mg finasteride and the untreated group. Moreover, both the 0.5 mg finasteride group and the orchiectomy group exhibited increased prostate artery resistance. Orchiectomy led to a significant reduction in androgen concentrations within 30 days. Ultimately, the researcher concluded that a dosage of 0.5 mg/kg of finasteride produced more effective prostate apoptosis and hemodynamic effects compared to other medical treatments, while orchiectomy resulted in prostate atrophy and notable endocrine changes in dogs with BPH [55]. Golchin-Rad et al. [72] evaluated the changes in serum CPSE, prostate-specific antigen, testosterone, dihydrotestosterone, and prostate volume during finasteride treatment in dogs with BPH. The results indicated that administration of finasteride resulted in a significant decrease in the concentrations of prostate-specific biomarkers (prostate-specific esterase, prostate-specific antigen), dihydrotestosterone, testosterone, and the overall volume of the prostate in the dogs over 1 month. Furthermore, there were no changes in hematological and serum biochemical factors during the administration of finasteride [72].

### 3.4. Phosphodiesterase-5 (PDE-5) Inhibitors

The selectivity of PDE-5 for cyclic guanosine monophosphate (cGMP) makes it an appealing intracellular target for enhancing cGMP second messenger activity, which provides various therapeutic benefits such as promoting cell survival, proliferation, and cellular protection. Inhibiting PDE-5 facilitates vasodilation and relaxation of smooth muscle, a mechanism that has been utilized in the treatment of patients with BPH [73]. Tadalafil is the only approved PDE-5 inhibitor for managing BPH. Notably, tadalafil can be administered either on its own or in combination with 5α-R inhibitors for patients dealing with BPH [74]. Derakhshandeh et al. [30] assessed the effects of tadalafil on experimentally induced BPH in dogs. The dogs received tadalafil (5 mg/day orally) for four consecutive weeks. The results indicated that the use of tadalafil, like other treatments for BPH such as castration, and antiandrogenic medications, is intended to diminish the growth and size of the prostate. In their study, ultrasonographic findings noted a decrease in prostatic serum factors (CPSE) suggesting that tadalafil may effectively reduce prostatic cell growth and mass. Furthermore, castration and the administration of tadalafil both led to a reduction in serum concentrations of dihydrotestosterone in dogs, with reductions of approximately 2-fold for castration and 1.2-fold for tadalafil. No side effects were observed based on hematological and biochemical parameters, as well as clinical examinations conducted over the 4 weeks [30]. Another study evaluated oxidative stress and inflammatory proteins in dogs with BPH following treatment with an antiproliferative agent, tadalafil. The results showed a decline in serum levels of glutathione peroxidase and superoxide dismutase in the treated dogs. However, the researchers concluded that tadalafil did not effectively manage oxidative stress and the inflammatory mediators associated with BPH in dogs [75].

### 3.5. New and Nonroutine Methods

A nonsteroidal vitamin D receptor (VDR) agonist, CH5036249, was tested in dogs as an animal model to assess its potential for treating BPH. The drug demonstrated effectiveness in a spontaneous BPH beagle model, during 11 months. The growth of the prostate gland was inhibited in two out of three dogs compared to the control group. Notably, significant atrophy of the glandular epithelium was observed in all treated dogs [76]. One study indicated that age-related reductions in blood supply to the lower urinary tract might contribute to the development of BPH, potentially influencing its pathogenesis. This specific study examined the effectiveness of pulsed electromagnetic field therapy (PEMF) in dogs to modify prostate blood flow and evaluate its impact on BPH. PEMF treatment was administered for 5 min twice a day over three weeks to 20 dogs affected by BPH. The results showed a significant average reduction in prostatic volume of 57% after the 3-week treatment period, with no negative effects on semen quality, testosterone levels, or libido. These findings suggest that PEMF may be an effective treatment for BPH in dogs, and the lack of side effects raises the possibility of its suitability for human applications as well [77].

Minimally invasive treatments such as prostate artery embolization, transurethral needle ablation, transurethral microwave thermotherapy, high-intensity focused ultrasound (HIFU), laser treatments, and intraprostatic injections in dog and cryoablation [78] have proven to be effective and practical in human medicine. However, further investigations are necessary to assess their applicability and effectiveness in veterinary medicine, particularly treating conditions like BPH in dogs. Exploring these advanced techniques could offer new therapeutic options for managing prostate-related issues in veterinary patients.

## 4. Conclusion

Dogs diagnosed with BPH are often asymptomatic; however, when clinical signs manifest, the most prevalent symptom is a serious to sanguineous urethral discharge. Various diagnostic tools, including physical examinations, imaging, and histopathology are essential for the accurate diagnosis of BPH. Treatment options for BPH are diverse and are typically recommended when a dog exhibits mild to severe symptoms or when these clinical signs adversely affect the dog's quality of life. In many instances, it is feasible to maintain fertility in dogs with this condition by selecting alternative therapeutic approaches instead of castration. These alternatives focus on managing symptoms and reducing prostate size while allowing the dog to remain fertile. Treatment options vary and can include castration as well as medical therapies such as finasteride and tadalafil, which aim to reduce prostatic size. However, castration is regarded as the definitive treatment, as it leads to a significant decrease in prostate size and alleviates the symptoms associated with BPH.

## Data Availability

The data that support the findings of this study are available from the corresponding author upon reasonable request.

## Nomenclature

BPH: Benign prostatic hyperplasia
DHT: Dihydrotestosterone
CPSE: Canine prostate-specific esterase
CT: Computed tomography
CEUS: Contrast-enhanced ultrasonography
CTP: Computed tomography perfusion
FNA: Fine-needle aspiration
MGA: Megestrol acetate
MPA: Medroxyprogesterone acetate
CMA: Chlormadinone acetate
DMA: Delmadinone acetate
GnRH: Gonadotropin-releasing hormone
5α-R: 5α-reductase inhibitors
PDE-5: Phosphodiesterase-5
cGMP: Cyclic guanosine monophosphate
VDR: Vitamin D receptor
